# Isolation and Characterization of Phenolic Compounds and Anthocyanins from Murta (*Ugni molinae* Turcz.) Fruits. Assessment of Antioxidant and Antibacterial Activity

**DOI:** 10.3390/molecules20045698

**Published:** 2015-03-31

**Authors:** Maria Paula Junqueira-Gonçalves, Lina Yáñez, Carolina Morales, Muriel Navarro, Rodrigo A. Contreras, Gustavo E. Zúñiga

**Affiliations:** 1 Departamento de Ciencia y Tecnología de Alimentos, Universidad de Santiago de Chile, Ecuador St. 3769, Estación Central, Santiago, 9170124, Chile; E-Mails: karo.morales@gmail.com (C.M.); muri.navarro@gmail.com (M.N.); 2 CECTA (Centro de Estudios en Ciencia y Tecnología de Alimentos), Universidad de Santiago de Chile, Obispo M. Umaña, 050 – Ed. de Alimentos, Estación Central, Santiago 9170201, Chile; E-Mail: lina.yanez@usach.cl; 3 Laboratorio de Fisiología y Biotecnología Vegetal, Departamento de Biología, Universidad de Santiago de Chile, Alameda, 3363, Estación Central, Santiago 9170023, Chile; E-Mail: rodrigo.contrerasar@usach.cl

**Keywords:** phenolics, anthocyanins, antibacterial activity, antioxidant activity, HPLC/ESI-MS, *Ugni molinae* Turcz.

## Abstract

Berry fruit consumption has become important in the promotion of human health, mainly due to their phenolic compounds, which have been associated with protection against different pathologies, as well as antimicrobial and other biological activities. Consequently, there has been a growing interest in identifying natural antioxidants and antimicrobials from these plants. This study aimed to characterize the phenolic chemical composition and anthocyanin profile of murta (*Ugni molinae* Turcz.) fruit, and to evaluate the antioxidant and antimicrobial activity of its extracts (ethanolic and methanolic). LC/MS of the ethanolic extracts showed the presence of three major compounds: caffeic acid 3-glu, quercetin-3-glu and quercetin, while in the methanolic acid extract they were cyanidin-3-glucoside, pelargonidin-3-arabinose and delphinidin-3-glucoside. The antioxidant activity of ethanolic extracts (DPPH^•^ and ORAC assays) was higher than that of methanol acid extracts or purified anthocynins. Furthermore, the methanol acid extract showed an inhibitory activity against the bacteria *E. coli* and *S. typhi* similar to that of standard antibiotics. The results suggest that the antioxidant activity of the ethanolic extract is regulated by the high content of phenolic compounds and the fruit’s characteristic color is due to the content of pelargonidin-3-arabinose and delphinidin-3-glucoside. The obtained results demonstrated the appreciable antioxidant and antibacterial activities, providing opportunities to explore murta extracts as biopreservatives.

## 1. Introduction

Berry fruit consumption has become important in the promoting of human health, mainly due to their phenolic compounds, which have been associated with protection against different pathologies (e.g., several types of human cancer), and because of their anti-inflammatory, gastroprotective, antimicrobial and other biological activities [1]. Consequently, there has been a growing interest to identify natural antioxidants and antimicrobials from these plants. Several edible Myrtaceae fruits including the Chilean berry “murta” (*U. molinae* Turcz.) have been shown to be good sources of polyphenolic antioxidants [2], comparable to those of blueberry [3].

Nowadays, it is essential to isolate, identify and characterize the phytochemicals of locally grown fruits as well as their antimicrobial activity in order to develop the fruits and plants as potential sources of antioxidant and therapeutic agents [4,5].

Phenolic compounds (PCs), including flavonoids and non-flavonoid types (phenylpropanoids, phenolic acids and cathechins), are of interest in food science because they affect the color quality, astringency and shelf stability of fresh and processed fruit products [6]. The bioactive properties of individual plant PCs are influenced by their structure, for example, antioxidant activity is greater in flavonoids that possess neighboring hydroxyl groups on their C phenyl ring [7]. Isolation and characterization studies of PCs, in order to understand the multiple mechanisms of antioxidant action, make their diverse group an interesting target in the search for phytochemicals that are of benefit to health and offer a possibility of extending the shelf life of lipid-rich foods [8].

Anthocyanins are a group of flavonoid compounds that are responsible for the colors of many flowers, vegetables, fruits and berries, providing attractive colors, such as orange, red and blue, thus being considered a valuable source of natural food colorants [9]. As a consequence of the social trend towards the consumption of natural products instead of synthetic ones, anthocyanins have received in the recent years increasing attention as natural colorants in food systems [10]. In addition, anthocyanins have attracted considerable interest due to their health benefits, including anti-cancer, anti-inflammatory and vasoprotective effects [11,12]. Among all common fruits and vegetables in the diet, berries, especially those with dark blue or red colors, have the highest content of anthocyanins and antioxidant capacities [13].

Murta (*U. molinae* Turcz.), a berry also known as murtilla, is a wild native plant occurring in the lowlands of the southern mountains of Chile (between the VII and X Region). This shrub produces a small globular fruit with a diameter of 0.7 to 1.3 cm used in homemade jams, syrups, jams and dessert liqueurs [14]. This is an aromatic species of the Myrtaceae family widely used in folk medicine as an analgesic for different types of pain and as an anti-inflammatory [15]. This plant has been historically appreciated because of the pleasant flavor of its edible fruits, and has been introduced into the UK and Australia for ornamental purposes. Due to its perennial shrub properties and applications in food manufacturing, it has been gaining attention from Chilean farmers, who have already domesticated this plant on a large scale [16]. Murta fruit has created increasing commercial and scientific interest during recent years, since its chemical and organoleptic properties make it a product with clear exportation potentials [17]. Additionally, murta fruit is considered a valuable source of high quality pectin that has a chemical composition similar to that of commercial citrus pectin [18]. Several reports show an association between the antioxidant activity of murta fruits and leaves and the levels of polyphenols [3,19,20,21,22]. However, until now, the characterization of the phenolic compounds, including anthocyanin profile from this fruit and its antibacterial activity has not been reported.

In this context, the main objectives of the present study are: (a) characterization of phenolic compounds; (b) identification and quantification of anthocyanins; (c) determination of the *in vitro* antioxidant potential; (d) evaluation of the antibacterial activity of the methanol and ethanol extracts of murta fruits.

## 2. Results and Discussion

### 2.1. Identification of Phenolic Compounds of Murta Extracts

**Figure 1:**
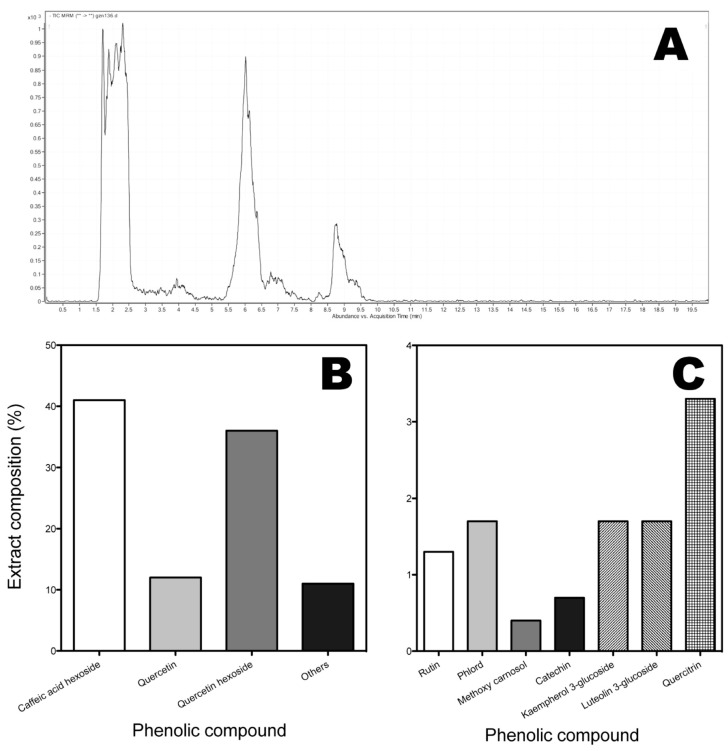
(**A**) Chromatogram of the relative content of phenolic compounds in murtilla fruits; (**B**) Profile of the main compounds as detected by LC/MS in MRM mode; (**C**) Profile of the minority compounds detected by LC/MS in MRM mode.

**Figure 2:**
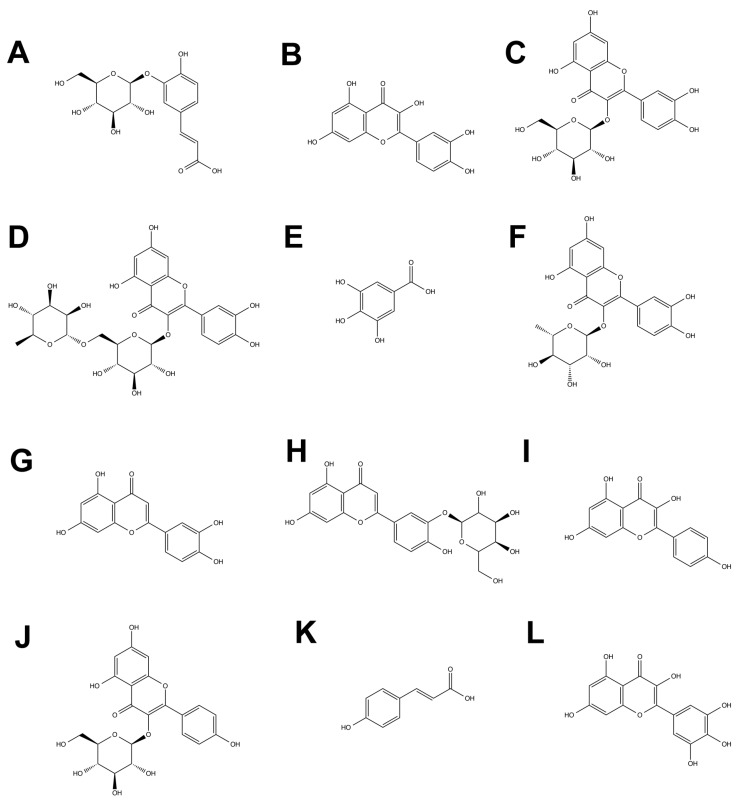
Structures of identified phenolics compouds in murta fruits. **A**: caffeic acid 3-*O*-glucoside; **B**: quercetin; **C**: quercetin 3-*O*-glucoside; **D**: rutin; **E**: gallic acid; **F**: quercitrin; **G**: luteolin; **H**: Luteolin 3-*O*-glucoside; **I**: kaempferol; **J**: kaempferol 3-*O*-glucoside; **K**: p-coumaric acid; **L**:myricetin.

The content of phenolic compounds obtained by LC/MS is shown in Figure 1. Twelve compounds were identified under the analytical conditions used, of which three constitute the major proportion: caffeic acid-3-glucoside, quercetin-3-glucoside and quercetin (Figure 1). The other compounds were rutin, gallic acid, quercitrin, luteolin, kaempferol, kaempferol-3-glucoside, luteolin-3-glucoside, *p*-coumaric acid and myricetin (Figure 1). Figure 2 shows the structures of these compounds, confirmed by comparison with authentic standards and MS fragmentation patterns reported in the literature. Previous studies have reported that fruits of murta contain flavonols, flavan-3-ols and anthocyanins [3].

Caffeic acid, one of the most common phenolic acids, frequently occurs in fruits, grains and dietary supplements for human consumption as simple esters with quinic acid or saccharides. Glycosylated derivatives of caffeic acid are responsible for different biological activities such as suppression of humoral and cellular immunity [23].

Quercetin is the main flavonol present in our diet [24]. Because of its interesting chemical and biological properties [25], quercetin has been one of the most studied flavonoids. In recent decades, many claims on the beneficial health effects of quercetin have been stated, including protection against various forms of cancer, cardiovascular diseases and neurodegenerative diseases [26]. Furthermore, anti-inflammatory, antibacterial and muscle-relaxant activities have been ascribed to quercetin [27]. Indeed, quercetin is known as a potent competitive inhibitor of certain cytochromes P450 [28] and sulfotransferases [29].

It has been suggested that quercetin is a potent anticancer agent in man [30]. Quercetin is also a strong antioxidant that can contribute to the prevention of atherosclerosis [31]. Quercetin has been shown to reduce the carcinogenic activity of several cooked food mutagens, enhance the anti**-**proliferative activity of anticancer agents, and inhibit the growth of transformed tumorigenic cells [32]. Currently, kaempferol is of interest because of its antioxidant [33], anti-tumor, anti-inflammatory, and anti-ulcer activity [34].

Quercitrin is a compound with important antioxidant and antibacterial effects [35,36]. Along with rutin, it may protect the cells of the colon [37]. An important effect of quercitrin is in decreasing the phototoxicity of hypericin [35], a substance from *Hypericum perforatum* L. that is similar to the buckwheat phototoxic substance fagopyrin.

### 2.2. Identification and Quantification of Anthocyanins by HPLC/ESI-MS

**Figure 3:**
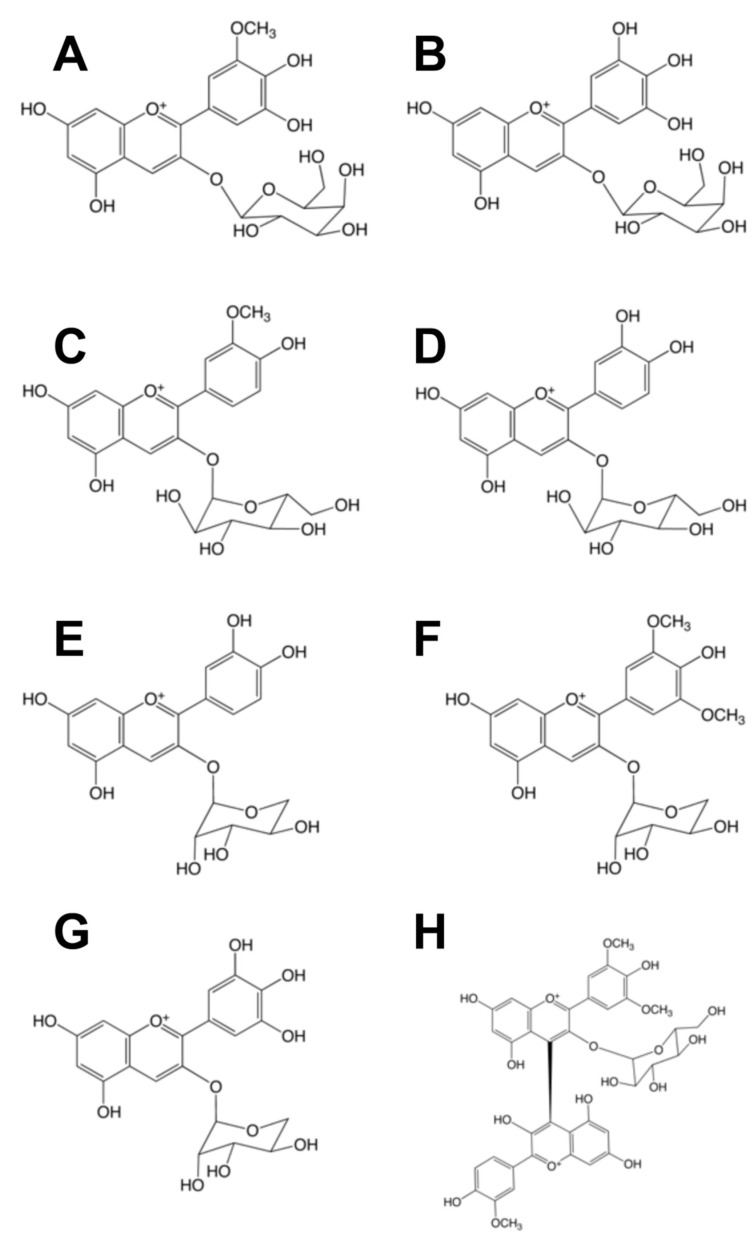
Structures of identified anthocyanins in *U. molinae* fruits. **A**: petunidin 3-*O*-galactoside; **B**: delphinidin 3-*O*-galactoside; **C**: peonidin 3-*O*-glucoside; **D**: cyanidin 3-*O*-glucoside, **E**: cyanidin 3-*O*-arabinoside; **F**: malvidin 3-*O*-arabinoside; **G**: delphinidin 3-*O*-arabinoside; **H**: peonidin-malvidin 3-*O*-glucoside.

The anthocyanin composition of murta berries was determined by HPLC/ESI-MS. Ten compounds were identified, based on HPLC retention time, elution order and ESI mass spectrometric data supported by comparison with our anthocyanin library and literature data [38]. Some general chromatographic trends were observed from this data. First, the general order of elution of anthocyanins under our analytical conditions was delphinidin, cyanidin, petunidin, malvidin and finally, peonidin. More specifically, the order of elution for a specific aglycone group was dependent on the attached glycoside; the order being galactoside < glucoside < arabinoside < rutinoside. By comparing the *m/z* values of each anthocyanin molecule and its fragmentation to the values in available published works, 10 peaks were tentatively identified in both kinds of extracts (Figure 3, Table 1). Data acquisition in the MRM mode was used for anthocyanin quantification (relative content) in the murta extracts.

**Table 1:** Main anthocyanins detected in murta fruits extracts by using LC/MS.

Peak No	Molucular Ion (*m/z*)	Fragment (*m/z*)	Compound	Proportion of each Crude Extract	Compound (%) Pure Extract
**1**	465	303	Delphinidin 3-*O*-glu	40.0	73.0
**2**	479	371	Petunidin 3-*O*-glu	6.0	5.0
**3**	463	301	Peonidin 3-*O*-glu	0.7	3.0
**4**	463	331	Malvidin 3-*O*-glu	0.4	0.1
**5**	449	287	Cyanidin 3-*O*-glu	0.4	4.3
**6**	449	287	Cyanidin 3-*O*-gal	0.5	2.6
**7**	435	303	Delphinidin 3-*O*-ara	3.6	10.0
**8**	519	271	Pelargonidin 3-*O*-ara	49.0	1.0
**9**	419	287	Cyanidin 3-*O*-ara	ND	1.0
**10**	549	271	Peonidin-malvidin 3-*O*-glu	2.0	3.0

**Figure 4:**
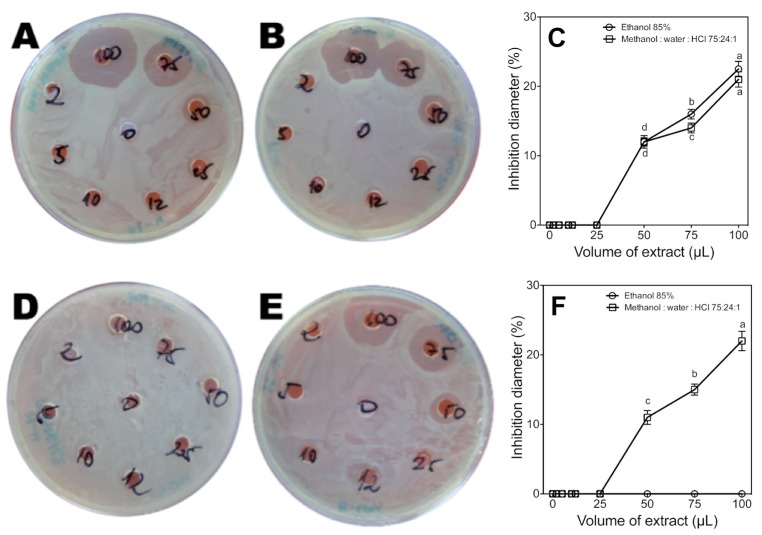
Antibacterial activity of extracts against *E. coli* (**A**,**B**) and *S. typhi* (**D**,**E**). The activity was measured in ethanolic extract (A,D) and acid methanol extract (B,E). **C** and **F** graphically represent the respective activities of *E. coli* and *S. typhi*. The results were analyzed by ANOVA with Tukey’s post test (*n* = 3; *p* < 0.05).

In crude acid ethanolic extract of murta, the main compounds found were pelargonidin-3-arabinose and delphinidin-3-glucoside with proportions of 49% and 40%, respectively. In the purified sample, the main compound detected was delphinidin-3-glucoside (73%) (Table 1). The individual chromatograms for anthocyanins in both extracts (MRM mode) were used to identify and quantify the anthocyanins listed in Table 1. Figure 4 shows the structure of the main anthocyanins identified in murta fruits; the predominant anthocyanin family contained in murta fruits was that formed by the glucose or arabinose derivatives of delphinidin, petunidin and cyanidin.

The total concentration of anthocyanins was not correlated with antioxidant activity, which could be attributed to the fact that glycosides (anthocyanins) are generally less active than aglycones (anthocyanidins). It is important to note that when the extract was purified in order to obtain a fraction rich in anthocyanins, only one compound strongly predominated, with the remainder of those present in the crude extract markedly decreased (Table 1).

Anthocyanins are bioactive food compounds with a double interest, one technological, due to their impact on the sensorial characteristics of food products, and the other their health related properties through different biological activities, for example their effect on cardiovascular disease risk protection [39]. Previous works had shown that murta leaf extracts contain hydroxybenzoic acids, flavan-3-ols, glycosylated flavonols [20] and triterpene acids [40]. The analgesic properties of murta infusion are associated with both flavonoid glucosides and triterpenoids [41]. Recently, Rubilar *et al.* [22] have reported an analysis of leaf extracts of murta, describing as the main components hydrobenzoic acid derivatives, quercetin dirhamnoside and myricetin rhamnoside, and several others not identified. The difference in term of compounds, between our work and that reported by Rubilar *et al.* [22] can be explained by the method of obtaining the extracts. Rubilar *et al.* [22] used a 50% ethanol solution, whereas in this work we used an 85% ethanol solution. The compounds extracted from plant material vary depending on the polarity of the solvent. In addition, Rubilar *et al.* [22] used dried plant material (fruit, leaves and stems). It has been reported that drying affects the quality of the extracts and consequently its biological activity [42].

Given the importance of murta in the economy of rural communities in Chile, the description of the components present in fruits should help to add value to products produced from this species. In addition, the LC/MS methodology optimized in this work may be useful in quality control of standardized murta extracts.

### 2.3. Antioxidant Capacity

Antioxidant capacity, total anthocyanins and phenolic compounds of murta extracts are shown in Table 2. DPPH^•^ (antioxidant capacity) discoloration percentages of the crude extracts (ethanolic) were higher than for the methanol acid or purified extract. The antioxidant capacity of extracts was not significantly correlated with total anthocyanin content (Table 2). The content of total anthocyanins was higher in the purified extract than in the crude extract. The antioxidant capacity of the crude extract correlated with the content of phenolic compounds (Table 2). Several authors have shown that phenolic compounds are often involved in the antioxidant capacity of plant extracts [43,44,45]. In addition, the ORAC value measured for murta extracts was higher in the ethanolic extract than in methanol acid or purified extracts. Furthermore, the ORAC values for murta extract is higher than those reported for blueberries [46].

**Table 2:** Antioxidant capacity, percentage discoloration of DPPH^•^ consumed and ORAC value, total anthocyanin and phenolic compound content (mg·g^−1^ fresh weight) of murta.

	Antioxidant Activity		Total Anthocyanin (mg·g^−1^ fresh wt)	Total Phenolic (mg·g^−1^ fresh wt)
	% DPPH	ORAC Value (µmol Trolox/g)		
Total extract	68 ± 4	3300 ± 231	26 ± 4	210 ± 12
Purified extract	33 ± 5	406 ± 28	35 ± 2	-

### 2.4. Antibacterial Activity

The antibacterial activity of the extracts was assessed at different doses against *E. coli* and *S. typhi* (both Gram negative and common food contaminants [47]), and the results compared with the activity of some positive control antibiotics (Table 3). In the case of *S. typhi* the activity of 100 µL of methanolic extract was equivalent to the activity of all antibiotics tested (zone of inhibition = 22.5 mm), while for *E. coli*, the activity of 100 µL of extract was equivalent to the activity of tetracycline, amikacin, cefuroxim, cefotaxim ampicillin and ciprofloxacin (Table 3; Figure 4; *p* < 0.05).

**Table 3:** Summary of antibiotics against S. typhi and *E. coli*. The activity was registered in inhibition halo diameter (mm). All measurements are the mean of three independent experiments.

Antibiotic	*E. coli*	*S. typhi*
Tetracycline	28.3 ± 0.9	23.7 ± 0.4
Clotrimazole	11.0 ± 0.6	22.7 ± 0.7
Gentamicin	10.7 ± 0.3	24.3 ± 0.3
Amikacin	20.7 ± 0.3	24.0 ±.0.3
Ceftriaxone	17.7 ± 0.9	23.3 ± 0.7
Cefuroxim	20.7 ± 0.7	24.3 ± 0.3
Cefotaxim	19.3 ± 0.7	24.3 ± 0.3
Ampicillin	22.3 ± 0.9	22.7 ± 0.3
Ciprofloxacin	22.3 ± 0.9	23.7 ± 0.9
Ampicillin/Sulbactam	12.3 ± 0.7	23.7 ± 0.3

The contradictory effectiveness of leaf extracts of *U. molinae* has previously been reported [48,49]. There is no report about the antibacterial effect of murta fruit extracts. According to the demonstrated activity against *E. coli* and *S. typhi* the results support the use of this plant as a food additive due to its antimicrobial activity.

Resistance to antimicrobial agents has become an increasingly important and pressing global problem. Various plant extracts possess bacteriostatic and bactericidal effects due to the secondary metabolites they contain, namely alkaloids, tannins, flavonoids and phenolic compounds. Most of these secondary metabolites, other than possessing antimicrobial potential, can also act as potent antioxidants [50].

## 3. Experimental Section

### 3.1. Chemicals and Solvents

Ethanol, methanol and acetonitrile HPLC grade were purchased from J.T Baker Chemical Co. (Phillipsburg, NJ, USA). Phosphoric and hydrochloridric acid analytical grade were purchased from Riedel-de-Haëhn (Seelze, Germany). 1-diphenyl-2-picrylhidrazyl (DPPH^•^), 2,4,6-tris(2-pyridyl)-*s*-triazine (TPTZ), FeCl_3_•6H_2_O, sodium acetate and HPLC standards (gallic acid, quercetin, rutin, chlorogenic acid, caffeic acid, syringic acid, myricetin, *p*-coumaric acid, ellagic acid, kaempferol, naringenin, isoquercitrin, morin, genistein and luteolin), red pyrogallol, 2,2'-azobis(2-amidinopropane) dihydrochloride (AAPH) and Trolox were purchased from Sigma-Aldrich Chemical Co. (St. Louis, MO, USA). Müller-Hinton agar was purchased from Merck (Darmstadt, Germany).

### 3.2. Plant Material

The ripe berries of murta (*Ugni molinae* Turcz.), genotype 14-4, were manually harvested and obtained from INIA Carillanca, Murta Gene Bank (Project FONDEF D05I1086), located in Puerto Saavedra (latitude, 38°45'S; longitude, 73°21'W), Chile, during summer 2009. Because the experimental field is located in a coastal area near to the Pacific Ocean, the weather is characterized as being a moderate oceanic climate with marine influence. Fruit from 5.5-year-old murta plants drip irrigated periodically during spring and summer, were used in the experiment. The fruits were placed in polyethylene bags and stored at −20 °C until use.

### 3.3. Extraction of Phenolic Compounds and Anthocyanins

Ten g of fresh fruit was mixed with 85% ethanol (100 mL, 85 v of absolute ethanol and 15 v of pure water) and then sonicated for 2 h. The extract was subsequently filtered through Whatman No. 2 filter paper. The final resulting solution was used as a stock solution for analyzing total phenolic content and profile, and the antioxidant and antibacterial activity.

Anthocyanins were extracted according to the methodology described by Fuleki and Francis [51]. One gram of fruit was macerated with ethanol (5 mL, 70% v/v acidified with 0.1% v/v of HCl), and the juice was stirred in the dark for 4 h and stored at 5 °C for 24 h. The samples were filtered through gauze and the solid residues were submitted to the same procedure, adding 10 mL of the extracting solvent. The resulting extract was completed with the extracting solvent to a volume of 30 mL.

### 3.4. Purification of Anthocyanins

Five milliliters of the anthocyanin aqueous solutions obtained from the extraction procedure were passed through an open column (20 mL) filled with ion exchange resin (Dowex Monosphere 88) in a sodium form. Anthocyanins were absorbed onto the column while sugars, acids and other water**-**soluble compounds were removed by washing the columns with 5 volumes of distilled water and 2.5 volumes of methanol. Anthocyanins were then eluted with methanol acidified with HCl in the following sequence: 7.5 volumes of methanol acidified with 0.1% HCl (v/v), 7.5 volumes of methanol acidified with 0.2% HCl (v/v), 7.5 volumes of methanol acidified with 0.3% HCl (v/v), 12.5 volumes of methanol acidified with 0.5% HCl (v/v), 12.5 volumes of methanol acidified with 1.0% HCl (v/v). To determine the volumes of solvent used and to control the releasing of anthocyanins from the column, spectrophotometric readings were carried out at 470 and 535 nm for each 10 mL eluted. The acidified methanolic fractions containing anthocyanins were combined and concentrated to 5 mL using a rotary evaporator at 38 ± 1 °C, then read in the spectrophotometer and frozen at −20 °C for further analysis of identification.

### 3.5. Analysis of Phenolic Content

Phenolic compounds in the ethanol extracts of murta were analyzed according to the methodology of Bravo *et al.* [52]. LC/MS analyses were performed using an Agilent 1100 series liquid chromatograph/mass selective detector equipped with a quadrupole (G6400) mass spectrometer (Agilent Technologies, Palo Alto, CA, USA). The liquid chromatographic system consisted of a quaternary pump, on-line vacuum degasser, and thermostatic column compartment, connected in line to a mass spectrometer. Data acquisition and analysis was carried out in an Agilent Mass Hunter. Sample (10 µL) was injected into the HPLC system, and separation was performed on a Nucleosil 120 C18 reversed phase column (250 × 4.6 mm i.d., 5 µm particle size) protected with an ODS RP18 guard column. A binary gradient of 1% formic acid in deionized water (solvent A) and acetonitrile (solvent B) was as follows: from 10% to 20% solvent B over 20 min, 20% to 25% solvent B over 10 min, 25% to 30% solvent B over 10 min. and then isocratically for 10 min. The flow rate was 1 mL/min. The mass spectrometer was fitted to an atmospheric pressure electrospray ionization (ESI) source, operated in negative ion mode. The electrospray capillary voltage was set to 3000 V, with a nebulizing gas flow rate of 9 L/h and a drying gas temperature of 300 °C. Mass spectrometry data were acquired in the Scan mode (mass range *m*/*z* 100–1000) and MRM mode.

### 3.6. Chemical Identification of Anthocyanins

Anthocyanins in the purified and crude extracts were separated by reverse phase HPLC on a Zorbax Eclipse XDB-C18 column (5 μm, 4.6 × 250 mm, Agilent Technologies, Santa Clara, CA, USA). The column was eluted with a mobile phase consisting of H_2_O:methanol:formic acid (75:20:5, v/v/v) with flow rate of 0.5 mL/min. The separated anthocyanins were further identified by LC-MS (Agilent) employing gel electrospray ionization (ESI) and operating in a triple quadruple mode. The instrument was scanned over the *m/z* range of 100 to 1000 in the ESI positive ion mode. The LC-MS was eluted with acetonitrile (ACN) and 0.5% NH_4_OH (90:10, v/v) with a flow rate of 0.4 mL/min. A binary solvent system was employed, following the work of Qin *et al.* [53]. Solvent A was 5% aqueous formic acid (v/v) and solvent B was 100% methanol (HPLC grade). The flow rate was maintained at 0.8 mL/min. The gradient elution profile was as follows: 0 min, 20% B; 1–10 min, 20%–27% B; 10–15 min, 27%–33% B; 16–20 min, 33%–20% B.

### 3.7. Analysis of Antioxidant Capacity (DPPH^•^ and ORAC Assays)

DPPH^•^ was dissolved in 85% ethanol [54] and the experiments were performed on freshly prepared solution. The assay conditions were as follows: 20 µL of each extract and 980 µL of DPPH^•^ solution (64 µM). Absorbance was recorded in a UV-visible spectrophotometer (Agilent model 5420, Palo Alto, CA, USA) at 517 nm during an interval from 10 s to 4 min of reaction. The results were expressed as % of consumed DPPH^•^.

The ORAC value was estimated according to Alarcon *et al.* [55]. Briefly, a solution containing PGR (5 µM), AAPH (10 mM), with and without the extract sample (ethanolic or methanolic), was incubated at 37 °C in phosphate buffer 75 mM, pH 7.4. PGR consumption was evaluated from its absorption intensity (A) decrease at 540 nm. UV-visible experiments were carried out in a Hewlett Packard 8453 spectrophotometer (Palo Alto, CA, USA). Control solutions (in the absence of extracts, A0) showed a fast consumption of the probes (less than 20 min). Values of (A/A0) were plotted as a function of time. Integration of the area under the curve (AUC) was performed up to a time such that (F/F0) reached a value of 0.2. These areas were employed to obtain ORAC values, according to Equation (1). All experiments were carried out in triplicate.

ORAC= [(AUC − AUC°)/(AUC_trolox_ − AUC°)] × f_[Trolox]_ (1)

where:

AUC = Area under curve in the presence of the tested extract sample, integrated between time zero and that corresponding to 80% of the probe consumption;
AUC° = Area under curve of control (PGR or FL plus AAPH solution);
AUC_trolox_ = Area under curve in the presence of Trolox;
f = Dilution factor, equal to the ratio between the total volume of the working solution (target molecule plus AAPH, plus extract) and the added extract sample volume;
[Trolox] = Trolox milimolar concentration.

### 3.8. Antibacterial Activity of Extracts

The antibacterial activity of the extracts was measured on strains of *Escherichia coli* (ATCC 25922) and Salmonella enteric serovar typhi (*S. typhi*) (ATCC 14028). Both strains were grown in Mueller-Hinton broth at 37 °C to saturation (OD600 = 0.6). For antibiotic analysis, 100 µL of saturated broth were poured onto plates with 15 mL of Mueller-Hinton agar medium and dispersed using a microbiological rake. Different aliquots of ethanolic and acidic methanolic extracts were evaluated (100, 75, 50, 25, 12, 10, 5 and 2 µL). The results were expressed as an inhibition diameter using the vehicle as a negative control. All samples were analyzed in triplicate.

### 3.9. Statistical Analysis

Determinations were based on two replicates in triplicate. Data statistical analyses were carried out by one-way ANOVA using the SPSS software (version 11.0, IBM Inc., Chicago, IL, USA). Significantly different means were compared using the multiple comparisons test of Tukey (*p* < 0.05).

## 4. Conclusions

LC/MS of ethanolic extracts of murta fruits showed the presence of three major compounds: caffeic acid-3-glu, quercetin-3-glu and quercetin, while the acidic methanol extract showed the presence of pelargonidine 3-arabinose, delphinidin-3-glucoside and petunidin-3-glucoside as major compounds. Our data suggested that the antioxidant and antibacterial activity shown by the ethanolic extract is regulated by the high content of phenolic compounds, and the characteristic color of fruits is mainly determined by the content of pelargonidine 3-arabinose and delphinidin-3-glucoside. The antioxidant activity of ethanolic extracts (DPPH^•^ and ORAC assays) from murta fruit was higher than the activity of crude or purified anthocynins. Ethanolic and methanolic extracts of murta showed a strong antibacterial activity. The results provide opportunities to explore this fruit as biopreservatives due to its antimicrobial and antioxidant activity.
